# The Changing Gender Differences in Life Expectancy in Chinese Cities 2005-2010

**DOI:** 10.1371/journal.pone.0123320

**Published:** 2015-04-13

**Authors:** Yan Le, Jie Ren, Jie Shen, Tong Li, Cheng-Feng Zhang

**Affiliations:** 1 Department of Dermatology, Huashan Hospital, Fu Dan University, Shanghai, China; 2 Department of Epidemiology, Key Laboratory of Public Health Safety of Ministry of Education, Public Health, Fudan University, Shanghai, China; UCL, UNITED KINGDOM

## Abstract

**Objectives:**

To analyze the gender difference in life expectancy in Chinese urban people and explore the age-specific and cause-specific contributions to the changing gender differences in life expectancy.

**Methods:**

Data of life expectancy and mortality were obtained from “Annual statistics of public health in China.” The gender difference was analyzed by decomposition method, including age-specific decomposition and cause-specific decomposition.

**Results:**

Women lived much longer than men in Chinese urban areas, with remarkable gains in life expectancy since 2005, respectively. The gender difference reached a peak in 2007. Mortality difference between men and women in the 60–79 age group made the largest contributions to the gender gap in life expectancy in all 6 years. Among causes of death, cancers, circulatory diseases and respiratory diseases made the largest contributions to the gender gap. 33–38% of the gender gap were caused by cancers, among which lung cancer contributed 0.6 years of the overall gap. The contribution of cancers to the gender gap reduced over time, mostly influenced by the narrowing effect of liver cancer on gender gap. Traffic accidents and suicide were the external causes influencing the gender gap, contributing 10–16% of the overall difference.

**Conclusion:**

Public health efforts to reduce excess mortalities for cancers, circulatory disease, respiratory diseases, and suicide among men in particular might further narrow the gender gap in life expectancy in Chinese cities.

## Introduction

Women generally had longer life expectancy at birth than men over the past two centuries while life expectancy increased steadily in both two genders [1,2]. However, the male-to-female life expectancy gender gap varied across time and country. Gaps were relatively small in the late 19th century but grew rapidly throughout the 20th century [3]. It is worthwhile to note that in recent years, a narrowing trend of the gap has been observed in many developed countries [4].

In China, great improvements have been achieved in life expectancy of both men and women during the past several decades [5,6–11]. However, specific data about life expectancy or its gender gap among Chinese population are still unavailable. With the data from life expectancy in Chinese cities of 2010, our study focused on the current status of life expectancy and the gender difference from 2005 to 2010. The objective of our study is therefore to explore which age groups and causes of deaths have contributed the most to the changes of the gender gap in life expectancy in Chinese cities, so as to find out major contributing factors of this gap variation, explore solutions for further improving life expectancy and reducing gender differences, and provide fundamental data and constructive suggestions for policy-formulators of public health.

## Materials and Methods

### Data

Life table and annual mortality data were obtained from *Annual statistics of public health in China* [6–11] from 2005 to 2010. The journal provides renewed data about health condition of Chinese residents and development of Chinese health, which is officially released by National Health and Family Planning Commission of the People’s Republic of China. It collected statistic data of residents’ health condition from 31 provinces (autonomous regions and municipalities) throughout the nation. Most of the data came from annual health statistics report and some part from sampling survey. A life table was drawn and life expectancy at birth for men and women was calculated annually. Causes of death were coded according to the International Classification of Diseases, 10th Revision (ICD-10) for all years using the conversion table published by WHO. We analyzed 35 specific causes of death (see Table 1) with relevance to the gender difference.

**Table 1:** Life expectancy at birth in Chinese cities and the gender gap between 2005 and 2010.

year	Life expectancy (male)	Life expectancy (female)	Gender gap
2005	74.28	79.09	4.81
2006	76.14	81.00	4.86
2007	76.42	81.72	5.30
2008	77.74	82.43	4.69
2009	77.20	82.02	4.82
2010	75.74	80.65	4.91

Since the data used in our study is publicly available, no ethical permissions are involved.

### Statistical analysis

Life expectancy is a summary of age-specific mortality rates. Therefore the difference in life expectancy between any two groups (e.g. male and female) can be interpreted by the formula of mortality difference at specific ages. For each specific age group, we used Arriaga’s method [12–15] to estimate how many years of the overall gender gap in life expectancy at birth are due to gender differences in age-specific mortality rates. We decomposed the changes of life expectancy (total effect) into three parts: direct effect, indirect effect and interaction. Given the additivity of total effect among varied ages, we analyzed the possible effects on the gender differences of life expectancy in different age groups, so as to predict contributions the age-specific mortality rate made to the total gender differences in life expectancy at birth.

The effect (in years) is estimated as:

$${}_{n}\Delta_{x}=[\frac{l_{x}^{M}}{l_{0}^{M}}\times (\frac{{}_{n}L_{x}^{F}}{l_{x}^{F}}-\frac{{}_{n}L_{x}^{M}}{l_{x}^{M}})]+[\frac{T_{x+n}^{F}}{l_{x+n}^{F}}\times \frac{\frac{l_{x}^{M}l_{x+n}^{F}}{l_{x}^{F}}-l_{x+n}^{M}}{l_{x}^{M}}]$$

_*n*_Δ_*x*_ refers to the effect of mortality differences between men and women on gender difference in life expectancy at birth from age *x* to *x+n* in a given year. *l*_*x*_ refers to the number of survivors to age x out of a synthetic cohort (*l*_*0*_ is the cohort size, normally 100,000 in a period life table); _*n*_ *L*_*x*_ refers to the number of person-years from age *x* to *x+n*; *T*_*x+n*_ refers to the number of total person-years alive from age *x*. The superscripts *M* and *F* denote “male” and “female”, respectively.

This equation decomposed the effects by two parts:

The first part is the direct effect of gender differences on the life years between age *x* and *x+ n*. The direct effect is the proportion of men who have lived up to the age x in living cohort, multiplied by the differential of average person-years between men and women living from age *x* to *x+n*.

For a particular age group between *x* and *x+n*, the lower the age-specific mortality of women is, the more contribution to life expectancy at birth is made at the age *x+n*. As a result, even if no difference is achieved in those groups beyond age *x+n*, the difference between age *x* and *x+n* still affects the total gap.

However, it is observed that the age-specific mortality of men and women differs in all age groups so that there is always an additional interaction effect due to the additional female survivors which will be exposed to new mortality conditions in the later periods.

The second part includes indirect effect and interaction. This part is also the proportion of the remaining female life expectancy at age *x+n* over the whole extra survivals in the living cohort. Indirect effect is the effect of having extra survivors even if mortality in other age groups remains the same, while interaction is the effect of having those survivors meeting different mortality conditions in subsequent age groups. The oldest age group (survivors over 80s), only has a direct effect.

In addition, every age group is to some extent also affected by causes of death. Given that the effect on life expectancy by any cause of death in any age group is equivalent to the effect on the total age-specific mortality by any cause of death, the contributions of cause-specific differences on male-or-female mortality differences from age *x* to *x+n* can be estimated with:

$${}_{n}\Delta_{x}^{i}{=}_{n}\Delta_{x}\times \frac{{}_{n}p_{x}^{i,F}{-}_{n}p_{x}^{i,M}}{{}_{n}r_{x}^{F}{-}_{n}r_{x}^{M}}$$

_*n*_ *P* ^*i*,*f*^_*x*_ is the female mortality between age *x* and *x+n* caused by disease *i*. _*n*_ *P* ^*i*,*m*^_*x*_ is the male mortality between age *x* and *x+n* caused by disease *i*. _*n*_ *r* ^*f*^_*x*_ is the total female mortality between age *x* and *x+n*. _*n*_ *r* ^*m*^_*x*_ is the total male mortality between age *x* and *x+n*.

Therefore, with a specific cause of death, the gender gap is affected by the dual proportion of the absolute gender difference in age-specific mortality and the gender difference related to other diseases.

The equation indicates that the total gender gap of life expectancy is widened by the causes of death occurring more frequently in men and narrowed by those occurring more in women.

In some particular periods, the cause-specific contributions to the gender gap of life expectancy are also the differences of the effects at the beginning and the end of this period. Even when mortality caused by a certain factor decreases both in men and women, the differences in different decreasing rates will nevertheless alter the total male-to-female life expectancy gap differences.

## Results

### Changing trends of life expectancy and its gender gap in China

**Fig 1:**
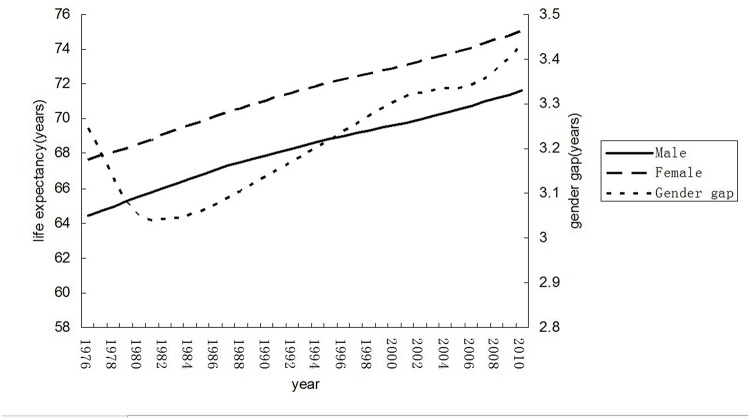
Trends of life expectancy at birth in China and the gender gap between 1976 and 2010.

Great improvements have been achieved in life expectancy among men and women over the past decades in China, with a relatively steady progress annually since 1970 (Fig 1). The gender differences in life expectancy were presented in an irregular curve: keeping declining to the bottom until 1980 and bouncing remarkably with a minor fluctuation in 2005.

### Life expectancy and its gender gap in Chinese cities from 2005 to 2010

As indicated in table 1, life expectancy in Chinese cities increased continuously since 2005. For men, it rose from 74.28 years in 2005 to 77.74 years in 2008, while for women, the number rose from 79.09 to 82.43, accompanied by a slight drop in both genders between 2009 and 2010. Women always enjoyed an extra life expectancy advantage over men throughout all these years.

With regard to the gender difference in life expectancy, this gap stayed comparatively steady during 2005–2006 but showed an apparent peak in 2007, when the difference reached 5.3 years, and then decreased to the bottom in 2008, at a difference of 4.69 years. Not until the year 2010 did the gender gap return to its original level displayed in 2005.

### Age-specific contributions to gender difference in life expectancy in Chinese cities

**Fig 2:**
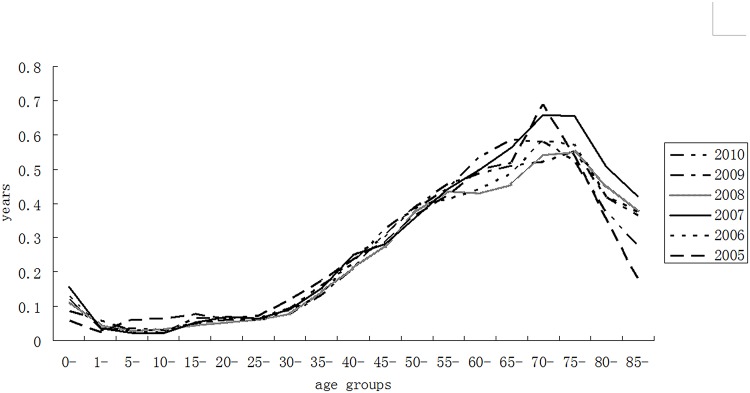
Age-specific contributions to the gender gap in life expectancy in Chinese cities between 2005 and 2010.

Fig 2 depicts the trends of age-specific gender differences between 2005 and 2010. Among all age groups, the most distinct mortality difference came from age group 60–79, making the largest contributions (roughly 42%-47%) to the total observed gender differences of life expectancy. As the life expectancy at birth of both men and women kept rising, the the age group beyond 80 years had an increasing impact on the gender gap, which contributed 0.53 years (11.05%) in 2005, then rose to 0.82 years (17.61%) and 0.92 years (17.46%) in 2007 and 2008, respectively, and finally ended up with 0.64 years (13.19%) in 2010.

Table 2 illustrates the trend of age-specific gender gap in life expectancy between 2005 and 2010. The values in the table display the direct effect of male-to-female mortality difference in each age group on the change of the overall gender gap of life expectancy. Positive values show the increase of the gender gap, while negative values imply narrowing ones.

**Table 2:** Age-specific contributions to changes in the gender gap in life expectancy between 2005 and 2010 (Note: positive values indicate widening the gender gap in life expectancy attributable to specific age groups and negative values indicate narrowing the gender gap).

Age groups	2005–2007	2007–2008	2008–2010
0-	0.099009	-0.04522	-0.02474
1-	0.012981	0.008147	0.013879
5-	-0.03935	0.005403	0.000805
10-	-0.04188	0.00999	-0.00909
15-	-0.02689	-0.00814	0.022471
20-	0.006134	-0.01762	0.016236
25-	-0.00772	-0.00286	0.004135
30-	-0.02923	-0.01199	0.016146
35-	-0.02178	-0.01346	0.019808
40-	0.013619	-0.03924	0.014848
45-	-0.00529	-0.00682	0.051278
50-	-0.00586	0.013261	0.011718
55-	0.0207	-0.00901	0.007647
60-	-0.00018	-0.06844	0.103532
65-	0.043027	-0.10843	0.132548
70-	-0.03043	-0.11552	0.037784
75-	0.115859	-0.10603	-0.02556
80-	0.153375	-0.05577	-0.0784
85-	0.242374	-0.04476	-0.10104
Total	0.498459	-0.61652	0.21399

According to the table, the gender gap in life expectancy increased 0.214 years between the year of 2008 and 2010. The age group of 60–70 made the largest contribution, with 0.23 years of increase. In this age group, the female life expectancy had a considerable increase, which directly widened the overall gender gap. Negative values appeared in three age groups, namely 0–1 year, 10–15 years and beyond 75 years, from 2008 to 2010, indicating the narrowing gender difference in life expectancy among the three groups between 2008 and 2010. This implied that men had an extra increase of life expectancy than women in these three age groups from 2008 to 2010.

Between 2007 and 2008, the overall gender gap shrank. Age-specific analysis further indicated that gender difference in life expectancy among all groups made negative contributions except the age groups 1 to 15 and 50 to 55. In the age group beyond 60 years, male life expectancy had a comparatively larger increase and exceeded female’s by 0.49 years.

The gender gap during 2005–2007 increased 0.50 years. The positive value indicated a widening gender gap and a larger increase of female life expectancy than male. Female life expectancy increased particularly fast in the infant group of 0–1 and the older group above 75 years, and women enjoyed an extra life expectancy advantage over men by 0.61 years.

### Cause-specific contributions of gender difference in life *expectancy*

Table 3 showed the contributions of major causes of death to gender difference in life expectancy between 2005 and 2010. Cancer, circulatory and respiratory diseases were the main causes of death which contributed the most to the gender gap of life expectancy in Chinese cities during the whole 6 years.

**Table 3:** Cause-specific contributions to the gender gap in life expectancy between 2005 and 2010.

Disease (ICD-10)	2010		2008		2007		2005	
	years	%	years	%	years	%	years	%
**Infectious diseases**	0.113	2.31	0.100	2.12	0.117	2.22	0.125	2.59
Respiratory TB	0.045	0.91	0.034	0.72	0.041	0.78	0.061	1.27
Other TB	-0.001	-0.03	0.000	0.00	0.002	0.04	0.002	0.04
Virus hepatitis	0.038	0.76	0.048	1.03	0.051	0.96	0.052	1.07
AIDS	0.007	0.14	0.004	0.09	0.004	0.07	0.001	0.01
**Cancer**	1.638	33.35	1.709	36.44	2.019	38.08	1.702	35.38
Malignant tumor	1.628	33.16	1.698	36.20	2.000	37.75	1.706	35.46
Nasopharyngeal carcinoma	0.025	0.52	0.037	0.78	0.048	0.90	0.027	0.57
Esophagus cancer	0.189	3.84	0.197	4.19	0.225	4.25	0.221	4.59
Stomach Cancer	0.232	4.73	0.239	5.10	0.312	5.88	0.280	5.82
Colorectal cancer	0.084	1.71	0.096	2.04	0.106	2.01	0.060	1.26
Liver cancer	0.428	8.72	0.406	8.66	0.503	9.50	0.562	11.68
Lung cancer	0.623	12.68	0.643	13.72	0.763	14.39	0.595	12.38
Mammary cancer	-0.155	-3.16	-0.174	-3.70	-0.173	-3.27	-0.133	-2.76
Cervical carcinoma	-0.063	-1.28	-0.040	-0.86	-0.043	-0.80	-0.039	-0.80
Carcinoma of urinary bladder	0.037	0.75	0.050	1.07	0.047	0.88	0.035	0.72
Leukocythemia	0.037	0.76	0.033	0.70	0.033	0.62	0.018	0.36
**Endocrine, nutritional & metabolic diseases**	0.013	0.26	-0.002	-0.05	-0.003	-0.05	-0.030	-0.63
Diabetes	0.010	0.21	0.003	0.07	0.005	0.09	-0.026	-0.54
Other endocrine	0.002	0.03	-0.006	-0.12	-0.008	-0.15	-0.001	-0.01
**Cardiovascular disease**	1.606	32.70	1.387	29.57	1.365	25.76	1.271	26.41
Acute rheumatic fever	-0.002	-0.03	-0.001	-0.03	0.001	0.03	-0.003	-0.07
Heart disease(Total)	0.706	14.37	0.613	13.08	0.592	11.16	0.504	10.48
Chronic rheumatic heart disease	-0.014	-0.29	-0.020	-0.42	-0.022	-0.42	-0.033	-0.70
Hypertensive cardiopathy	0.038	0.78	0.010	0.21	0.012	0.22	0.010	0.21
Acute myocardial infarction	0.287	5.85	0.322	6.86	0.239	4.50	0.181	3.77
Other coronary heart disease	0.209	4.26	0.157	3.34	0.147	2.77	0.092	1.92
Cor pulmonale	0.093	1.88	0.040	0.86	0.092	1.74	0.179	3.72
Other hypertension disease	0.066	1.35	0.038	0.80	0.070	1.31	0.109	2.27
Cerebrovascular disease	0.814	16.57	0.709	15.12	0.685	12.93	0.652	13.55
Other CVD	0.021	0.43	0.028	0.59	0.017	0.32	0.009	0.18
**Respiratory system**	0.551	11.23	0.674	14.36	0.800	15.09	0.588	12.23
Pneumonia	0.126	2.57	0.095	2.02	0.088	1.65	0.036	0.75
Chronic lower respiratory disease	0.341	6.94	0.487	10.38	0.619	11.68	0.483	10.05
Other respiratory	0.070	1.42	0.075	1.60	0.082	1.54	0.054	1.12
**Digestive system**	0.193	3.93	0.171	3.65	0.185	3.50	0.251	5.21
Gastric and duodenal ulcer	0.016	0.34	0.019	0.42	0.018	0.34	0.025	0.51
Intestinal obstruction	0.011	0.23	0.007	0.16	0.007	0.13	0.002	0.05
Liver disease	0.129	2.63	0.116	2.47	0.121	2.28	0.187	3.90
Other digestive	0.036	0.73	0.028	0.60	0.039	0.73	0.037	0.77
**External causes**	0.603	12.29	0.497	10.60	0.579	10.93	0.759	15.78
Traffic accident	0.168	3.43	0.159	3.38	0.000	0.00	0.232	4.83
Accidental fall	0.072	1.47	0.042	0.90	0.054	1.02	0.096	2.00
drawn	0.072	1.47	0.051	1.09	0.060	1.13	0.124	2.58
Suicide	0.026	0.53	0.020	0.42	0.000	0.00	-0.001	-0.02
Residual	0.193	3.93	0.155	3.30	0.237	4.48	0.145	3.02
Total	4.910	100	4.690	100	5.300	100	4.810	100

Infectious diseases contributed 2.1%-2.6% to the gender gap in life expectancy between 2005 and 2010. Relatively, infectious diseases had stronger impact on the gender gap when the gender differences of life expectancy were narrowed, and a larger gender gap was accompanied by weaker effect of infectious diseases. During the 6 years in our study, the lowest life expectancy gender gap appeared in 2005, 2.59% (0.125 years) of the total gender gap were contributed by infectious diseases. This decreased to 2.12% (0.1 years) in 2008, when the gap reached its maximum.

The contribution of cancer to the observed gap was 33%-38% (1.638–2.019 years). With time passing by, the contribution tended to decline accompanied by the increasing gap. Among those malignant tumors, lung cancer made the largest contribution to gap, followed by liver cancer and gastric cancer. Every year nearly 0.6 years of gender gap in life expectancy was contributed by lung cancer.

25%-33% (1.2–1.6 years) of the difference was contributed by circulatory diseases. Meanwhile, with time passing by, circulatory diseases made larger contributions to the gender gap (from 26.41% (1.27years) in 2005 to 32.7% (1.606 years) in 2010) while life expectancy in both genders kept increasing. Among all circulatory diseases, cerebrovascular diseases made the largest contribution (12%-17% of the total gap), followed by acute myocardial infarction and coronary heart disease. In addition, the contribution grew with increasing life expectancy over time.

Respiratory diseases contributed 11%-15% to the total gap. The contribution rose as the gap increased. In 2005 and 2010, the gender difference in life expectancy was narrowed, and the contributions of respiratory diseases to the gender gap were 12.23% (0.588 years) and 11.23% (0.551 years), while in 2007 and 2008, when the gap was widened, respiratory diseases contributed 15.09% (0.8years) and 14.36% (0.674 years), respectively. Among all the respiratory diseases that caused death, chronic lower respiratory diseases contributed the most to the gender difference and the contribution declined slightly as the overall life expectancy rose. Chronic lower respiratory diseases contributed 10.05% (0.483 years) to the total gap, and dropped to 6.94% (0.341 years) in 2010. Pneumonia contributed the second most to the total gap.

Non-disease factors also contributed to the gender gap in life expectancy, including external factors (such as trauma and intoxication), making up 10%-16% of the total gender gap. The contribution declined as the gender difference increased. In 2005 and 2010 when the gender gap was narrowed, the external factors contributed 15.78% (0.759 years) and 12.29% (0.603 years), respectively. In 2007 and 2008, the gap was widened, yet the contribution of external factors dropped to 10.93% (0.579 years) and 10.60% (0.497 years). Among these external factors causing death, traffic accidents made the largest contribution (about 3%-4% (0.15–0.2years)) to the gap. Besides, suicide-contributed gender difference grew along with life expectancy. In 2005, suicide had negative contribution to the gender gap of life expectancy, implying that more men committed suicide than women. The contribution had turned positive since 2007 and got stronger over time.

## Discussion

Since the 21st century, with a more varied configuration of social, economic and political roles for women, mortality gradient for men and women showed fluctuation to some degree. Among all the risk factors, smoking was believed to be the main factor responsible for the narrowed gender gap in life expectancy [16–19]. In this study, we tried to explore the age-specific and cause-specific contributions to the gender difference in life expectancy during 2005–2010 in Chinese cities. Our main findings are as follows: 1) Women lived much longer than men in Chinese urban citizens, with remarkable gains in life expectancy since 2005, respectively. 2) Cancers, circulatory diseases and respiratory diseases made the largest contributions to the gender gap. 3) Traffic accidents and suicide were the external causes influencing the gender gap.

Some studies have claimed that mortality in people under 35 years old made little contribution to the narrowing of the gender gap in life expectancy [20]. In South Korea, life expectancy of people under 35 years old made 0.6 years (approximately 1/3) to the total narrowed gender gap annually [21]. In our study, a similar contribution of 0.5 years (1/10) annually was made by people under 35 years in Chinese cities.

In our study, we used cause-specific decomposition analysis to explore the importance and contributions of different systems or diseases during different period to the gender gap in life expectancy. In the years between 2005 and 2010, cancers made the largest contributions to narrow the gender gap. Every year about 1/3 of total gender gap could be explained by the male-to-female difference in cancer mortality. Among all the cancers, lung cancer made the majority contributions. Smoking is a main risk factor for lung cancer. Smoking was regarded as the largest contributor to the gender gap of life expectancy in many developed countries. It was reported that 40%-60% of the gender gap in Europe was contributed by smoking [18,20]. This indicates that in Chinese cities, quitting smoking may be of great help to narrow the gender gap of life expectancy. Banning cigarettes in public places and indoors and smoke prevention measures will be helpful to reduce the mortality caused by lung cancer, especially in women, which will in return improve men’s life expectancy and narrow the male-to-female gender gap of life expectancy.

In our study, we found that the contributions of cancers to the gender gap of life expectancy were decreasing as time went by. Among all the cancers, liver cancer made the largest contributions to the gender gap of life expectancy, whose contributions continuously dropped. This implied that in Chinese cities, male mortality caused by liver cancer decreased more quickly than that of female. The contribution of liver cancer was negative to the total gap. It is well-known that the main risk factors of liver cancer include excessive drinking and HBV infection. With the rising burdens of living and working in Chinese cities, more and more Chinese women started drinking alcohol. Alcohol abuse is now becoming a vital problem during cities’ development [22]. Investigation revealed that women’s drinking rate in China is 4.5%. Almost 29.5% of women practice drinking every day, and 5.8% of them reached high risk level [22]. As a result, alcohol-related diseases and their mortality rates are now increasing, contributing directly to female life expectancy as well as the gender differences. By contrast, 10%-30% of the gender gap was contributed by alcohol-drinking in Europe [16].

The contribution of circulatory system diseases to the gender gap of life expectancy in the year 2005 was 26.41%, and then rose to 32.70% (nearly 1/3) in 2010. Cause-specific decomposition demonstrated that it was all sorts of cardiac and cerebrovascular diseases that contributed to this rise. In many countries, coronary artery disease made the largest contribution to the gender difference [15,21,23–25]. Our study found that the contribution of cardiac diseases to the gap was widening, which was in accordance with other studies reported in Japan [25] and South Korea [21].

It is worth noting that the contributions of external factors such as trauma or toxication to narrow the gender gap were weakening continuously, with a drop from 15.78% in 2005 to 12.29% in 2010. Deaths caused by external factors such as trauma, toxication are mostly associated with occupational injuries, indicating the gender gap owing to occupational reasons is now diminishing.

The contribution of suicide to the gender difference was positive in 2005, and then weakened with the time until turned to negative in 2010. According to a report in *The Lancet* [26], the suicide rate in China was 2.3 times more than the international average. The population of suicide in China occupies 1/3 of the total number all over the world. Suicide is now becoming the most common cause of death among Chinese young people, and women suicide rate in China exceeded men by 25%. Our study showed that the contribution of suicide to the gender gap in 2010 was 0.53%, suggesting the declining of women suicide rate in Chinese cities was faster than that of men. This might come as a result of the higher suicide rate in women. With a higher basic value, the decline was comparatively steeper as well.

The patterns of gender gap and causes of death varied in different countries. Influenced by different causes of death, the gender gap of life expectancy changed even in the same epidemiological circumstances. For example, in some developed countries like Canada, Sweden and United States, the gender gap in life expectancy was narrowing constantly, while in Japan this gap was widening [27]. In Chinese cities, the gender gap was presented in varied curves with narrowing or widening trends during different period. It has been reported that the gender mortality difference caused by cardiovascular diseases made the largest contribution to the narrowed gap in Canada, United States, Britain, Germany, Italy, France and Japan [20]. Our study suggested that cancers contributed most in Chinese cities to the gender gap of life expectancy. The impact of traffic accidents, violence and injuries on human health is now getting more and more global attention. Their contributions to gender gap were negative in Japan, while positive in widening in some other countries [20,27]. In China, the contributions acted in a way of first widening and then narrowing to the gender gap in life expectancy.

However, our study has several limitations. First, the study time we focused on was relatively short, only from 2005 to 2010. Given more data collected in a longer term, better understanding about the changing trends of gender gap would be achieved. Meanwhile, our study only analyzed the differences in life expectancy by decomposition of age-specific and cause-specific contributions. This decomposition analysis cannot directly reflect the impact of mainstream factors such as economics, politics and life on the gender differences of life expectancy at birth. Based on more detailed data, a further specific study is expected to dive deeper into main contributing factors to the gender difference in life expectancy such as alcohol and smoking related diseases.

In conclusion, we speculate that public health efforts to reduce excess mortalities for cancers, circulatory disease and respiratory diseases, suicide, among men in particular might further narrow the gender gap in life expectancy in Chinese cities.
